# Two new species of the *Brachycephalus pernix* group (Anura: Brachycephalidae) from the state of Paraná, southern Brazil

**DOI:** 10.7717/peerj.3603

**Published:** 2017-07-27

**Authors:** Luiz F. Ribeiro, David C. Blackburn, Edward L. Stanley, Marcio R. Pie, Marcos R. Bornschein

**Affiliations:** 1 Escola de Ciências da Vida, Pontifícia Universidade Católica do Paraná, Curitiba, PR, Brazil; 2 Mater Natura—Instituto de Estudos Ambientais, Curitiba, Paraná, Brazil; 3 Florida Museum of Natural History, University of Florida, Gainesville, FL, United States of America; 4 Departamento de Zoologia, Universidade Federal do Paraná, Curitiba, Paraná, Brazil; 5 Instituto de Biociências, Universidade Estadual Paulista, São Vicente, São Paulo, Brazil

**Keywords:** Atlantic rainforest, Serra do mar, Conservation status, Montane forest, Micro-CT

## Abstract

We describe two new species of miniaturized toadlet in the *B. pernix* group of *Brachycephalus* (Anura: Brachycephalidae) from the Atlantic Forest of the state of Paraná, southern Brazil. The first new species is distinguished from all congeners by the pale red coloration from the head to the pelvic region, with sides of the body and thighs dorsally yellowish green. It is known only from the type locality in a cloud forest at altitudes ranging between 1,144–1,228 m a.s.l. The second species, although more closely related to *B. izecksohni*, is morphologically similar to *B. brunneus* in its overall brown coloration, but distinct from that species in the color of the iris (black with conspicuous golden spots, instead of entirely black). It was found on three mountains, at altitudes between 1,095–1,320 m a.s.l., and in vegetation types including cloud forest, montane forest, and secondary forest. The two new species exhibit neither vertebral fusions nor osteoderms, but one has both a distinct neopalatine and well-developed odontoids on the maxillae. We discuss the conservation status of both species.

## Introduction

The first species of *Brachycephalus* (Anura: Brachycephalidae) was described in the early 19th century—*B. ephippium* (Spix, 1824)—and the genus remained monotypic until Heyer et al. (1990) raised *B. ephippium* var. *nodoterga* Miranda-Ribeiro, 1920 to species rank. These slow beginnings could not anticipate the remarkable number of new species that would later be discovered, including 22 new species described over the past decade (Alves et al., 2006; Pombal Jr & Gasparini, 2006; Alves et al., 2009; Haddad et al., 2010; Napoli et al., 2011; Pombal Jr & Izecksohn, 2011; Garey et al., 2012; Clemente-Carvalho et al., 2012; Condez et al., 2014; Ribeiro et al., 2015; Pie & Ribeiro, 2015; Condez et al., 2016; Bornschein et al., 2016a; Guimarães et al., 2017). Most of these new species belong to the *B. pernix* species group, one of the three currently recognized species groups in the genus, which includes 15 described species (Bornschein et al., 2016a; Bornschein et al., 2016b) that share a bufoniform body shape and lack osteoderms (Ribeiro et al., 2015), namely *B. albolineatus* Bornschein et al., 2016a; *B. auroguttatus* Ribeiro, Firkowski, Bornschein & Pie, 2015; *B. boticario* Pie, Bornschein, Firkowski, Belmonte-Lopes & Ribeiro, 2015; *B. brunneus* Ribeiro et al., 2005; *B. ferruginus* Alves et al., 2006; *B. fuscolineatus* Pie, Bornschein, Firkowski, Belmonte-Lopes & Ribeiro, 2015; *B. izecksohni* Ribeiro et al., 2005; *B. leopardus* Ribeiro, Firkowski & Pie, 2015; *B. mariaeterezae* Bornschein, Morato, Firkowski, Ribeiro & Pie, 2015; *B. olivaceus* Bornschein, Morato, Firkowski, Ribeiro & Pie, 2015; *B. pernix* Pombal, Wistuba & Bornschein, 1998; *B. pombali* Alves et al., 2006; *B. quiririensis* Pie & Ribeiro, 2015; *B. tridactylus* Garey et al., 2012; and *B. verrucosus* Ribeiro, Firkowski, Bornschein & Pie, 2015.

In the present study, we describe two new species of *Brachycephalus* from the state of Paraná, southern Brazil and assign them to the *B. pernix* group. This discovery is part of a continuing effort to investigate montane anurans of southern Brazil (see Bornschein et al., 2015; Pie & Ribeiro, 2015; Ribeiro et al., 2015; Bornschein et al., 2016a; Bornschein et al., 2016b). These species are diagnosed through a combination of coloration patterns, morphological traits, genetic distances, and genealogical information from phylogenetic analyses (Firkowski et al., 2016).

## Material and Methods

Specimens collected for this study were anaesthetized and euthanized using 2% chloridrate lidocaine, fixed in 10% formalin, stored in 70% ethyl alcohol solution, and deposited in the Museu de História Natural Capão da Imbuia (MHNCI), Curitiba, state of Paraná, Brazil and in the Museu Nacional (MNRJ), Rio de Janeiro, state of Rio de Janeiro, Brazil. Collection permits for this study were issued by ICMBIO (10.500, 22470–2/1911426). In addition, we examined specimens deposited in the following Brazilian collections: Célio F.B. Haddad collection, Departamento de Zoologia, Universidade Estadual Paulista, Campus de Rio Claro, state of São Paulo (CFBH); MHNCI; Coleção Herpetológica do Departamento de Zoologia, Universidade Federal do Paraná, Curitiba, state of Paraná (DZUP); MNRJ; Museu de Zoologia da Universidade de São Paulo, São Paulo, state of São Paulo (MZUSP); and Museu de História Natural, Universidade Estadual de Campinas, Campinas, state of São Paulo (ZUEC). We also examined specimens in the Museum of Comparative Zoology at Harvard University, Cambridge, USA (MCZ). A list of the examined specimens is provided in the Appendix. All specimens in the type series are adults.

Measurements were made with a micrometric eyepiece attached to a stereomicroscope. Measurements abbreviations were as follows (Lynch & Duellman, 1997; Heyer et al., 1990): snout–vent length (SVL); head length, from tip of snout to angle of jaw (HL); head width—greatest width of head located between angles of jaw (HW); eye diameter (ED); nostril diameter (ND); interorbital distance, between anterior corners of the eyes (IOD); internostril distance, between inner margins of nostrils (IND); eye–nostril distance, from anterior corner of the eye to posterior margin of nostril (END); thigh length (THL); and crus length (CL). All measurements are indicated in mm. The sex of the specimens was determined by the presence of the linea masculinea, which is only found in males. The linea masculinea consists of bands of fibrous connective tissue located over the entire extension of the oblique muscles (Duellman & Trueb, 1986). This character is present in all *Brachycephalus* species of the *B. pernix* group analyzed in this study. The linea masculinea can be easily seen when the skin of the ventral region is cut. We used the classification of Brazilian vegetation proposed by the RADAMBRASIL project (*in* Veloso, Rangel-Filho & Lima, 1991) to characterize the habitats of each of the species. Altitudinal records were obtained after plotting the geographical coordinates of the lowest and highest altitudinal records in the field using Google Earth. We obtained approximate estimates of male density of one of the species as in Bornschein et al. (2016a). We slowly walked along a small trail that crossed the study area as a transect. We found distinction points along this transect where we could hear the calls of the study species. Along different days, we spent 3 h in each point, placing markings on the vegetation at the positions where we recorded a calling male. We then measured the extent of the sampling area (=the extent of the auditory sampling) and counted the number of markings per sampled area. We recognize that this is an imprecise estimate, but given the scarcity of data on basic natural history of most *Brachycephalus* species, we believe that this information is still valuable. Given that sequences for the 16S gene were available for one of the new species based on a previous study (Firkowski et al., 2016), we assessed its evolutionary divergence from closely related species using uncorrected p-distances (the percentage of different nucleotides between a pair of sequences) both within and between species.

High resolution computed tomography (CT) scans were produced for one paratype of each species, as well as one specimen of *B. ephippium* for comparisons, at the University of Florida’s Nanoscale Research Facility, using a Phoenix v|tome|x M (GE’s Measurement & Control business, Boston, MA, USA) with a 180 kv X-ray tube with a diamond-tungsten target and with the following settings: 60 kV, 175 mA, a one-second detector time, averaging of three images per rotation and a voxel resolution of 7.5–11.5 µm. Raw X-ray data were processed using GE’s proprietary datos|x software v 2.3 to produce a series of tomogram images. These Micro-CT image stacks were then viewed, sectioned, measured, and analyzed using VG StudioMax 3.0.3 (Volume Graphics, Heidelberg, Germany). Final figures were prepared with Photoshop and Illustrator (CS5; Adobe, San Jose, CA, USA).

For consistency, our descriptions follow the layout of our other recent descriptions of new species of *Brachycephalus* (Pie & Ribeiro, 2015; Ribeiro et al., 2015; Bornschein et al., 2016a). Our descriptions use the general osteological terminology of Trueb, Diaz & Blackburn (2011) and the terminology for the ilium by Gómez & Turazzini (2015), though we refer to the manual digits as I–IV rather than II–V to avoid confusion for most taxonomists. These descriptions loosely follow the organization of the detailed osteological description recently provided for *Brachycephalus darkside* (Guimarães et al., 2017). The electronic version of this article in Portable Document Format (PDF) will represent a published work according to the International Commission on Zoological Nomenclature (ICZN), and hence the new names contained in the electronic version are effectively published under that Code from the electronic edition alone. This published work and the nomenclatural acts it contains have been registered in ZooBank, the online registration system for the ICZN. The ZooBank LSIDs (Life Science Identifiers) can be resolved and the associated information viewed through any standard web browser by appending the LSID to the prefix http://zoobank.org/. The LSID for this publication is: The online version of this work is archived and available from the following digital repositories: PeerJ, PubMed Central and CLOCKSS.

## Results

### *Brachycephalus coloratus* sp. nov

Urn:lsid:zoobank.org:act:urn:lsid:zoobank.org:act:D45D8C67-0F74-459E-8FC6-048748ED254B

Figs. 1– 5

**Figure 1:**
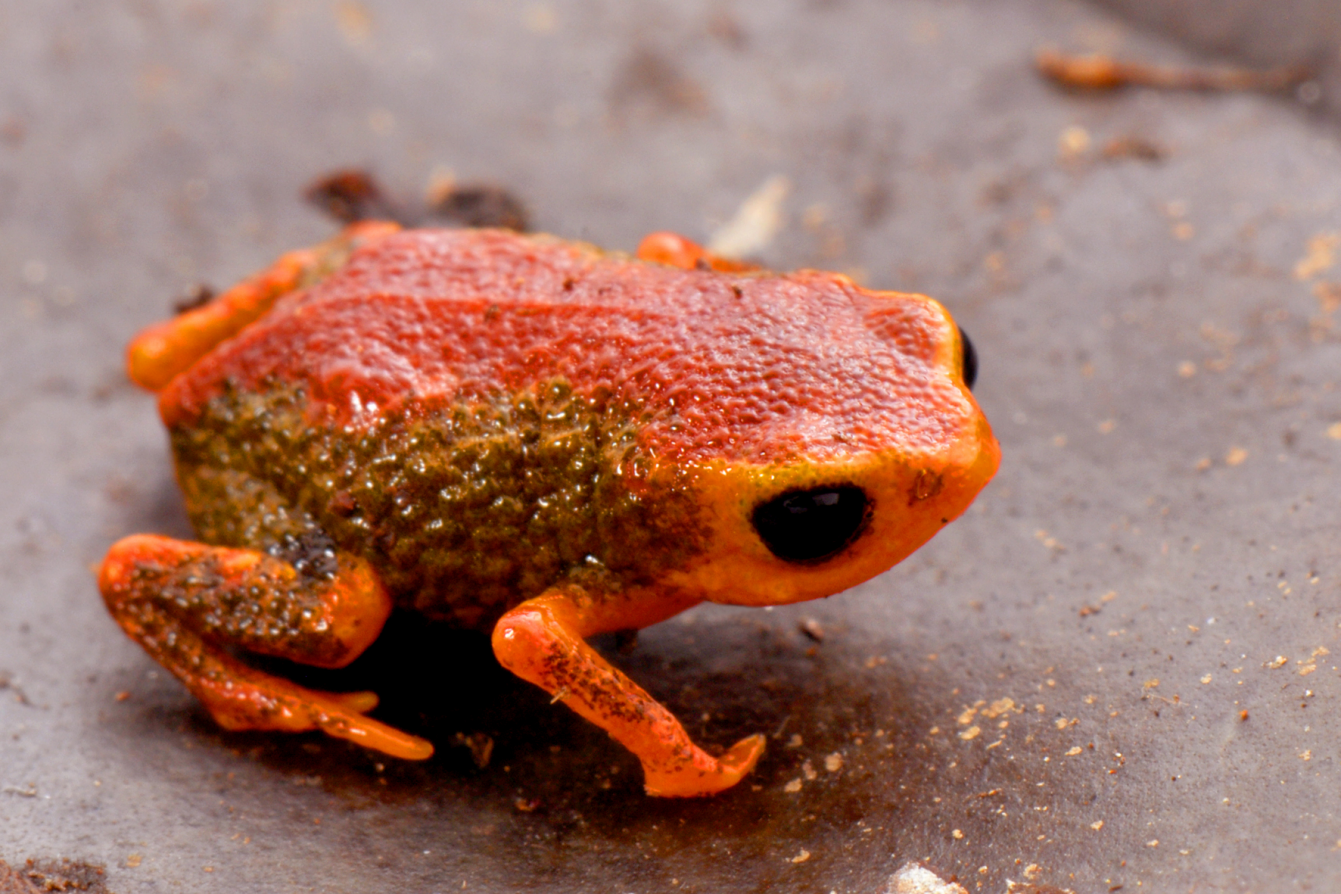
Holotype of *Brachycephalus coloratus* in life (MHNCI 10273).

**Figure 2:**
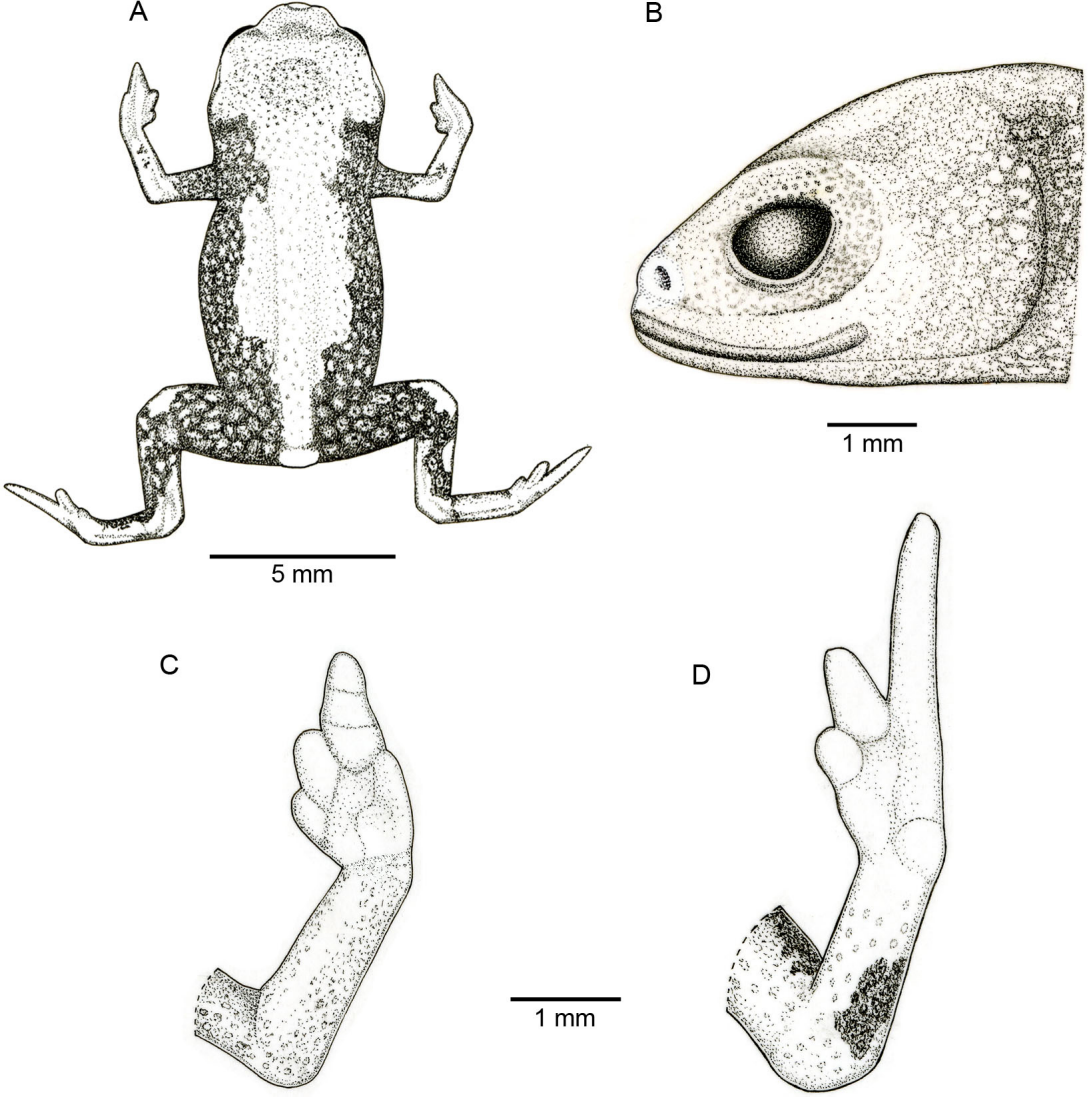
Holotype of *Brachycephaluscoloratus* (MHNCI 10273). (A) Dorsal view of the body, (B) lateral view of the head, (C) ventral view of right hand, and (D) ventral view of right foot. Drawing by Marcello Brotto.

**Figure 3:**
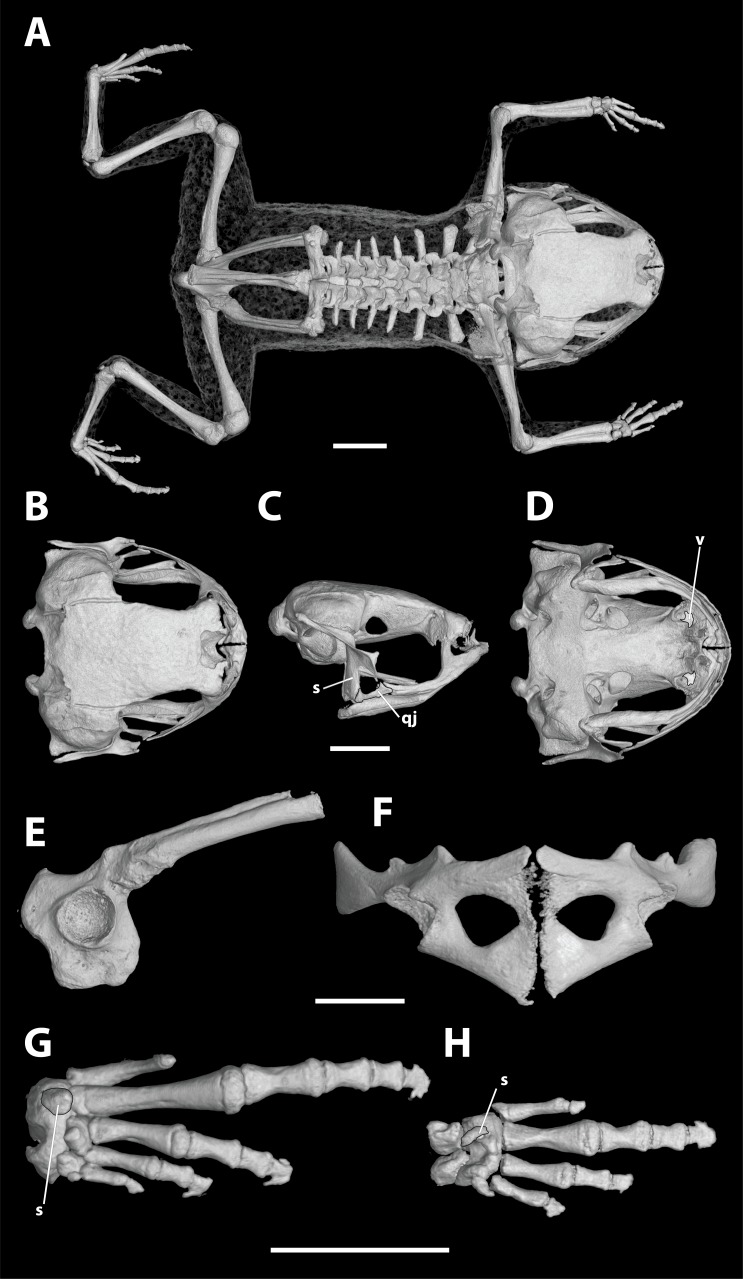
High-resolution computed tomography (CT) scans of a paratype of *Brachycephalus coloratus* (MHNCI 10274) showing key osteological features. While the vomer is fused to surrounding elements, we have highlighted its approximate boundaries. (A) Dorsal view of the skeleton; (B) dorsal, (C) lateral, and (D) ventral views of the skull (without the lower jaw); (E) ilium in lateral view; (F) pectoral girdle in ventral view; (G) right foot in plantar view; and (H) right hand in palmar view. Abbreviations: qj, quadratojugal; s, sesamoid; v, vomer. Scale bars equal 2 mm.

**Figure 4:**
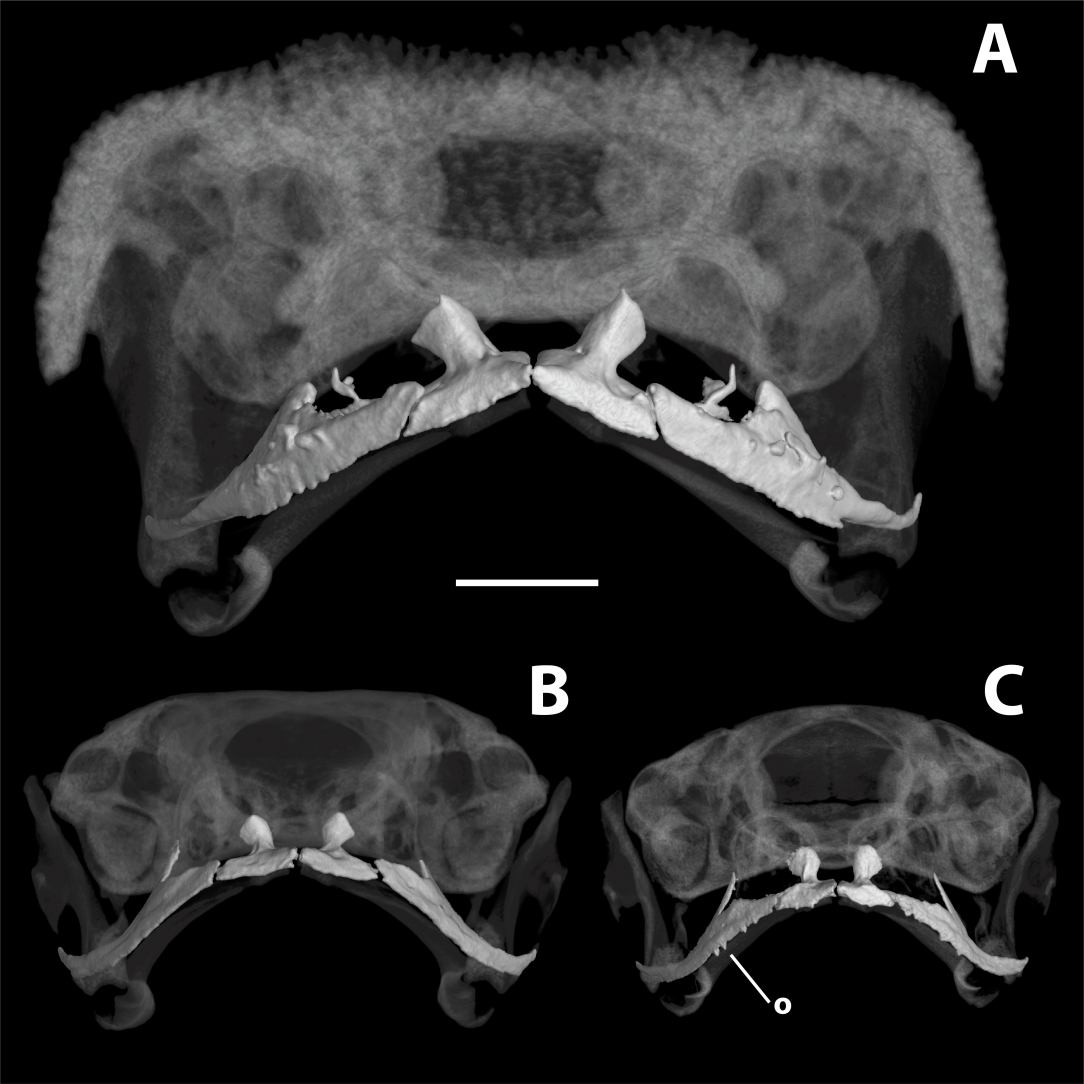
High-resolution computed tomography (CT) scans of *Brachycephalus* specimens in rostral view. Images provide comparisons of odontoids between (A) *B. ephippium* (MCZ A–108655), (B) *B. coloratus* (MHNCI 10274), and (C) *B. curupira* (MHNCI 10285). Abbreviation: o, odontoid. Scale bar equals 1 mm.

**Figure 5:**
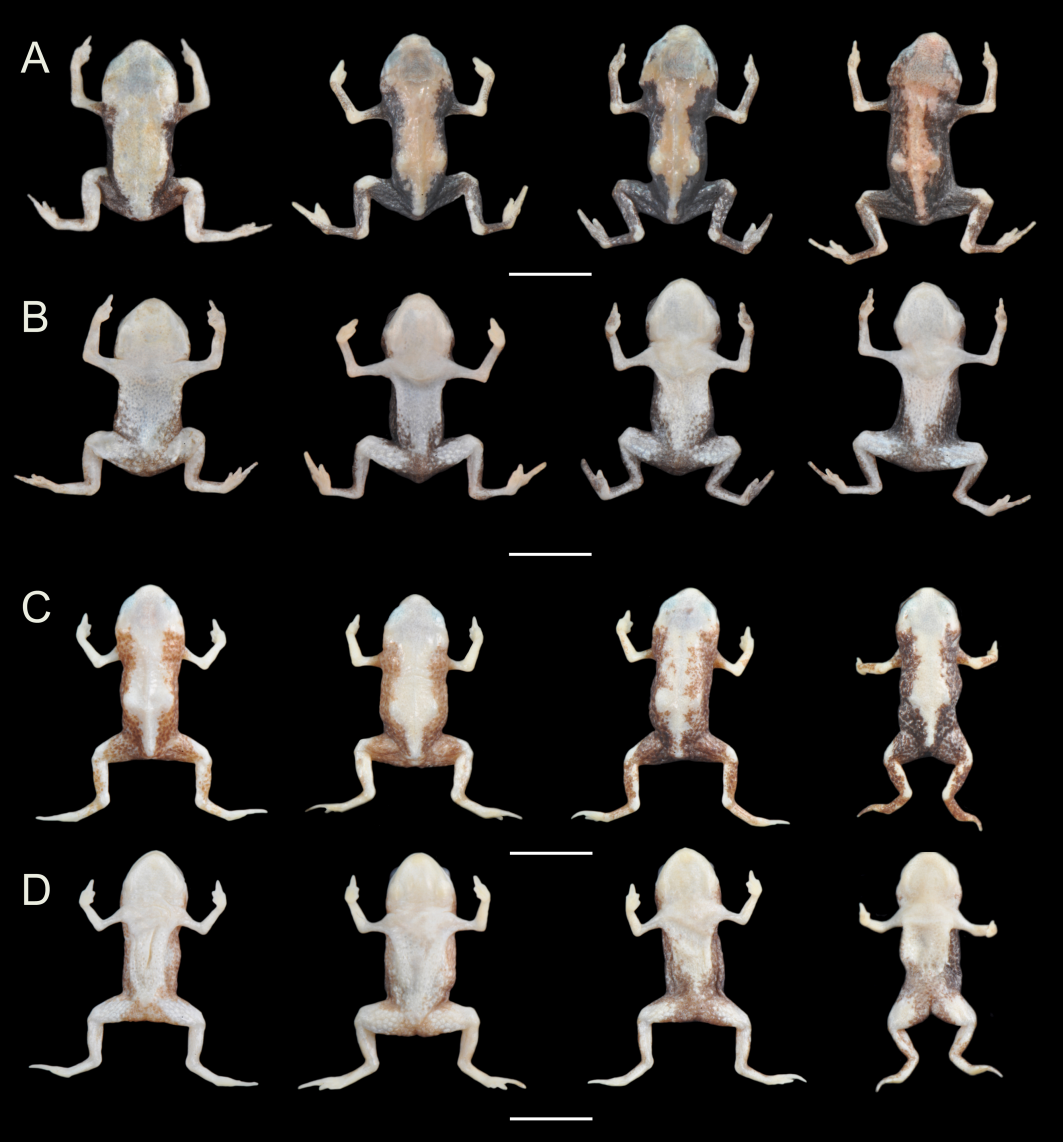
Variation in the coloration of preserved specimens of *Brachycephalus coloratus* and *B. pernix*. *Brachycephalus coloratus* ((A) dorsal view, (B) ventral view, from the left: MHNCI 10276, holotype, MHNCI 10275 and MHNCI 10277 paratypes) in contrast with the nearest congener, *B. pernix* ((C) dorsal view, (D) ventral view, from the left: MHNCI 9806-07, MHNCI 10157, MHNCI 10160). Specimens were chosen to represent the most extreme variation in our sample of preserved specimens.

**Holotype.** MHNCI 10273 (Figs. 1 and 2) adult male, one of a series collected at Estância Hidroclimática Recreio da Serra (25°27′14″S, 49°00′27″W; 1,144 m a.s.l.), Serra da Baitaca, municipality of Piraquara, state of Paraná, southern Brazil, on 1 October 2015 by Luiz Fernando Ribeiro and Marcelo Brotto.

**Paratypes.** MHNCI 10274 adult male, collected on 1 October 2015 by Luiz Fernando Ribeiro & Marcelo Brotto; MNRJ 89949–50 adult females, collected on 12 November 2015 By Luiz Fernando Ribeiro & Marcelo Brotto; MHNCI 10275 adult female, collected on 26 October 2015 by Luiz Fernando Ribeiro & Marcio Roberto Pie; MHNCI 10276 adult male, collected on 26 October 2015 by Luiz Fernando Ribeiro & Marcio Roberto Pie; MHNCI 10277–8 adult male, collected on 3 December 2015 by Luiz Fernando Ribeiro; MHNCI 10279 adult female, collected on 3 December 2015 by Luiz Fernando Ribeiro. The entire type series was collected between 1,144–1,228 m a.s.l.

**Diagnosis.** *Brachycephalus coloratus* is a member of the genus *Brachycephalus* based on diagnostic morphological traits, including phalangeal reduction, an arciferal pectoral girdle in which the ossified procoracoid and epicoracoid cartilages are fused to the clavicle, coracoid, and scapula, a suprascapula expanded with a prominent cleithrum, and the absence of a sternum (Bornschein et al. (2016a), and modified from Kaplan (2002); Izecksohn (1971); Ford & Cannatella (1993); Ribeiro et al. (2005); Alves et al. (2006); Silva, Campos & Sebben (2007); Fig. 3). *Brachycephalus coloratus* is a member of the *B. pernix* group, as defined by Ribeiro et al. (2015), by having a bufoniform body and lacking osteoderms. Within *Brachycephalus*, *B. coloratus* is distinguished from all of the species in the genus by the following combination of characters: (1) body robust and bufoniform; (2) adult size SVL 10.3–13.3 mm; (3) smooth dorsum (Fig. 1); (4) absence of osteoderms; (5) unornamented dermal skull bones that lack co-ossification to the skin; (6) lack of fusion among vertebrae (Fig. 3); (7) absence of distinct neopalatines and vomers (if present, joined by synostosis with sphenethmoid); (8) robust squamosal with anterior zygomatric ramus expanded dorsoventrally; (9) presence of quadratojugals; (10) weakly developed odontoids on the maxillae (Fig. 4); (11) general color (in life) of the dorsal region of the head pale red from the head to the pelvic region, and yellowish green along the sides of the body and dorsum of the thighs (Fig. 1); and (12) iris completely black (Fig. 1). *Brachycephalus coloratus* is unique among other members of its species group by the presence of three main colors (pale red from the head to the pelvic region; yellowish green on the lateral body and dorsal thighs; yellow chin and ventral arms and legs; Fig. 1), as opposed to the one or two main colors present in all remaining species in the *B. pernix* group. In preservative, the coloration of *B. coloratus* becomes similar to preserved *B. pernix*, but differs from that species by the sides of the body being dark brown, as opposed to a lighter brownish coloration with a reddish hue in *B. pernix* (Fig. 5). The smooth dorsum of *B. coloratus* is similar to that of *B. albolineatus*, *B. brunneus*, *B. leopardus*, *B. pernix*, *B. ferruginus*, *B. izecksohni*, *B. pombali*, and *B. tridactylus* (as opposed to the rugose dorsum of *B. auroguttatus*, *B. boticario*, *B. fuscolineatus*, *B. mariaeterezae*, *B. olivaceus*, *B. quiririensis*, and *B. verrucosus*). The new species lacks the osteoderms that characterize species of the *B. ephippium* group, as defined by Ribeiro et al. (2015), and the bufoniform shape and larger body size of the new species distinguish it from those in the *didactylus* group, as defined by Ribeiro et al. (2015), which are smaller (SVL = 8–10 mm) and have a leptodactyliform body shape.

**Description of the holotype.** Male with robust bufoniform body; head slightly wider than long; head length 36% of snout–vent length; snout short: its shape semicircular in dorsal view, and rounded in lateral view (Figs. 2A and 2B); nostrils protuberant, directed anterolaterally; canthus rostralis not distinct; lips nearly sigmoid; loreal region weakly concave; eye slightly protruding in dorsal and lateral view; eye diameter 33% of head length; tympanum indistinct; vocal sac not expanded externally; tongue longer than wide, with posterior half not adherent to floor of mouth; choanae relatively small, rounded; vomerine teeth absent.

Upper arm and forearm relatively slender, upper arm approximately as long as forearm; tip of fingers I and II rounded, tip of Finger III pointed; relative lengths of fingers IV < I < II < III; subarticular tubercles and inner and outer metacarpal tubercles absent; legs short, thigh robust; thigh length 37% of SVL, crus length 85% of thigh length; toes II–IV short, relatively distinct; toes I and V not visible externally; relative length of toes II < III < IV; subarticular tubercles and inner metatarsal tubercles absent; outer metatarsal tubercle distinct, large and ovoid.

Skin smooth on dorsum of head and central body, and lacking osteoderms; skin granular on dorsolateral surfaces of body, flanks, and dorsal surface of thighs, with juxtaposed, large glandular warts; sides of the body granular; large, round juxtaposed glandular warts on the sides of the body, belly and thighs; chin, arms, and legs smooth.

**Coloration of the holotype.** In life, dorsum pale red from head to pelvic region; sides of body and dorsum of thighs yellowish green; face, arms and remaining parts of legs bright yellow; chin and ventral sides of arms and legs yellow; belly yellow becoming light green in inguinal region; iris black (Fig. 1). In preservative, reddish region on dorsum becoming caramel, greenish region on sides and inguinal region of belly becoming dark brown, and yellowish green regions on flanks becoming dark brown (Fig. 5).

**Measurements of holotype (in mm).** SVL = 10.3, HL = 3.7, HW = 4.0, ED = 1.3, ND = 0.2, IOD = 2.1, IND = 1.2, END = 0.6, THL = 3.9, CL = 3.3.

**Variation in the type series.** Measurements (in mm) of five adult males are (mean ± SD, with range in parentheses): SVL = 10.4 ± 0.1 (10.3–10.6); HL = 3.7 ± 0.1 (3.6–3.9); HW = 4.1 ± 0.2 (3.9–4.4); ED = 1.2 ± 0.1 (1.2–1.4); ND = 0.2 ± 0.0 (0.2–0.2); IOD = 2.2 ± 0.1 (2.1–2.3); IND = 1.2 ± 0.0 (1.2–1.3); END = 0.6 ± 0.0 (0.6–0.7); THL = 3.8 ± 0.2 (3.6–4.1); CL = 3.3 ± 0.2 (3.1–3.6). Measurements of four adult females are: SVL = 12.9 ± 0.5 (12.2–13.3); HL = 4.3 ± 0.1 (4.1–4.4); HW = 4.9 ± 0.1 (4.9–5.0); ED = 1.4 ± 0.0 (1.4–1.4); ND = 0.2 ± 0.0 (0.2–0.2); IOD = 2.5 ± 0.1 (2.4–2.6); IND = 1.3 ± 0.1 (1.3–1.4); END = 0.7 ± 0.1 (0.7–0.8); THL = 4.4 ± 0.1 (4.3–4.6); CL = 3.5 ± 0.3 (3.0–3.7). There are slight differences in the extent of the pale dorsal coloration, which is evident even in preserved specimens (Fig. 5).

**Description of general osteology.** Based on Micro-CT scan of MHNCI 10274 (paratype; male; Fig. 3). *Brachycephalus coloratus* is broadly similar to that of other species in the genus. The skull is compact, slightly wider than long, and lacking ornamented dermal roofing bones. The well-developed parotic plate is synostosed to the frontoparietal approximately at the location of a prominent groove for the occipital artery that is closed at its posteriormost extent. The nasals, frontoparietals, prootics, exoccipitals, sphenethmoids, and parasphenoid are synostosed, contributing to well-defined and bony margins to both the optic fenestrae and prootic foramina. The premaxillae are broad, widely separated, and lack odontoids; each has a robust pars dentalis, and a robust alary process that is taller than wide and narrowly separated from the adjacent nasal. In ventral view, the maxillae are nearly straight and bear weakly developed odontoids. The quadratojugals are prominent with a broad articulation with the maxillae. The pterygoids are slender, each with a long anterior ramus that approaches but does not articulate with the adjacent maxilla, a short posterior ramus articulating with the ventral ramus of the squamosal, and a short and broad medial ramus articulating with the prootic. Distinct vomers and neopalatines are not observable and, if present, are synostosed to the surrounding sphenethmoid. Small, curved sphenethmoids are present at the anterior margin of the nasal capsule. The parasphenoid is broad and robust. The squamosals are robust, and each has a large rectangular zygomatic ramus that is expanded dorsoventrally at its anterior margin approaching the maxilla and a long and slender posterior ramus that has a broad connection to the prootic. Each fenestra ovalis is covered entirely by a robust, ossified operculum; a stapes (or columella) is not present. The posteromedial processes of the hyoid are ossified and slender. The arytenoid cartilages are strongly mineralized.

There are eight distinct, procoelous, imbricating presacral vertebrae that are not fused to one another. The neural spines of presacral IV–VIII are closely associated and appear interlocking in dorsal view. The atlas lacks transverse processes and has widely separated cotyles. The sacrum is procoelous with stout transverse processes, each of which has a dorsal tubercle at the distal third. A sesamoid at the distal transverse process of the sacrum is present at its articulation with the ilium. The urostyle is relatively stout with a tall dorsal ridge that decreases in height posteriorly.

The robust pectoral girdle is arciferal and formed by the synostosis of the clavicle, coracoid, scapula, and strongly mineralized epicoracoid and procoracoid cartilages. The scapula is stout and bears a prominent anterior process. A bony sternum and omosternum are not present.

The pelvic girdle is a robust element composes of synostosed ilium, pubis, and ischium. The circular acetabulum is completely ossified with well-defined margins. The shaft of the ilium stout and roughly straight in both lateral and dorsal views, and bears both a weakly developed dorsal protuberance and a weakly developed dorsal crest. There is a broad ventral acetabular expansion comprised of both the ilium and ossified pubis.

The forearm is slightly shorter than the humerus. The distal carpals (Element Y and II–V) are fused. The radiale and ulnare are large and subequal in size. The phalangeal formula for the manus is 1–2–3–1 and there is both a single ossified prepollex and a small palmar sesamoid. The tips of the terminal manual phalanges are arrow-shaped in digits I–III but blunt in IV. The tibiofibula and femur are similar in length. There are two large distal tarsals. The phalangeal formula for the pes is 1–2–3–4–0 and there is a single small ossified prehallux and a plantar sesamoid. The tips of the terminal pedal phalanges are arrow-shaped in digits II–IV but blunt in I and V.

Tomograms (tif format) and shape files (stl format) of the studied specimen are freely available at http://morphosource.org/Detail/SpecimenDetail/Show/specimen_id/4219.

**Etymology.** The specific epithet is from the Latin *coloratus* (“colored”, “variegated”) in reference to the unique combination of colors found in the species.

**Figure 6:**
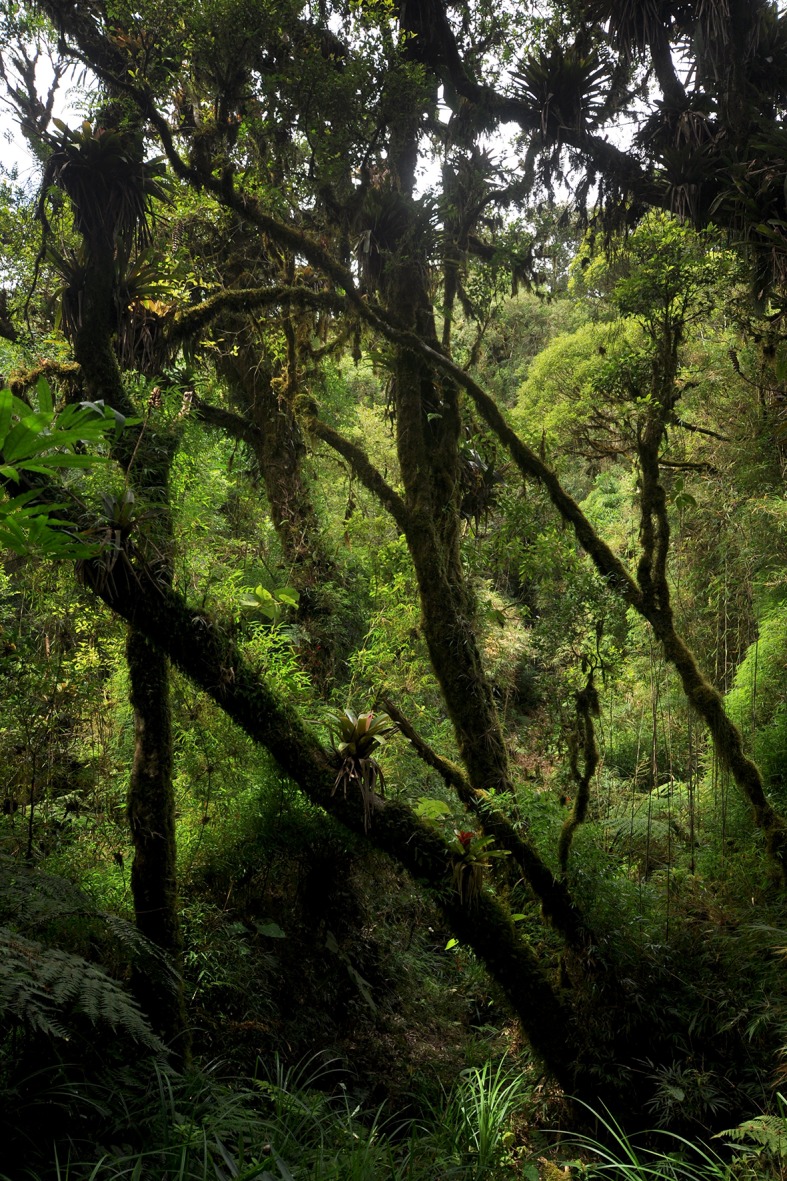
Vegetation at the type locality of *Brachycephalus* coloratus, at 1,144 m a.s.l. characterized by high-elevation forest (Floresta Ombrófila Altomontana).

**Distribution and habitat.** *Brachycephalus coloratus* is currently known from the type locality, living on the ground under the leaf litter. Additional fieldwork was carried out at the neighboring Pão de Ló mountain (25°24′35″S, 49°00′00″W; 1,115 m a.s.l.), which is distant 4.96 km from the type locality on a straight line, on 24 January 2016, but failed to provide evidence for the presence of the species. Another expedition to the same site in October 2015 also found no evidence of *Brachycephalus* at that location (SAA Morato, pers. comm., 2015). The habitat of *B. coloratus* is characterized by high-elevation forest (Floresta Ombrófila Densa Altomontana), but with some elements of montane forest (Floresta Ombrófila Densa Montana) below ∼1,180 m a.s.l., including many epiphytes (ferns and bromeliads; Fig. 6) and tree ferns (Cyatheaceae).

**Remarks.** The type locality of *Brachycephalus coloratus* is 7.3 km distant in a straight line from the type locality of *B. pernix* (both species occur in the Serra da Baitaca). This geographical proximity between them could indicate that they represent sister species. Indeed, preliminary genetic data seems to confirm this hypothesis (MJ Nadaline, pers. comm., 2016). More detailed mapping of their distributions are still necessary to determine the extent to which these species are allopatric.

The area of occurrence of *Brachycephalus coloratus* is within a land development (Estância Hidroclimática Recreio da Serra), more specifically in a region where a road is planned to be constructed, which raises severe concerns regarding the preservation of the species. On the other hand, contacts with a local resident indicated that areas above 1,000 m a.s.l. will not be occupied, despite the original plans when the enterprise was approved by the municipality. Furthermore, we obtained record of the new species at 130 m from the border of a state park—the Parque Estadual da Serra da Baitaca—and the potential occurrence of the species in this park should be a priority to design a conservation initiative. We suggest that the species should be considered as Data Deficient (*sensu* IUCN, 2012) given the limited available information.

### *Brachycephalus curupira* **sp. nov.**

**Table:** 

**Figure 7:**
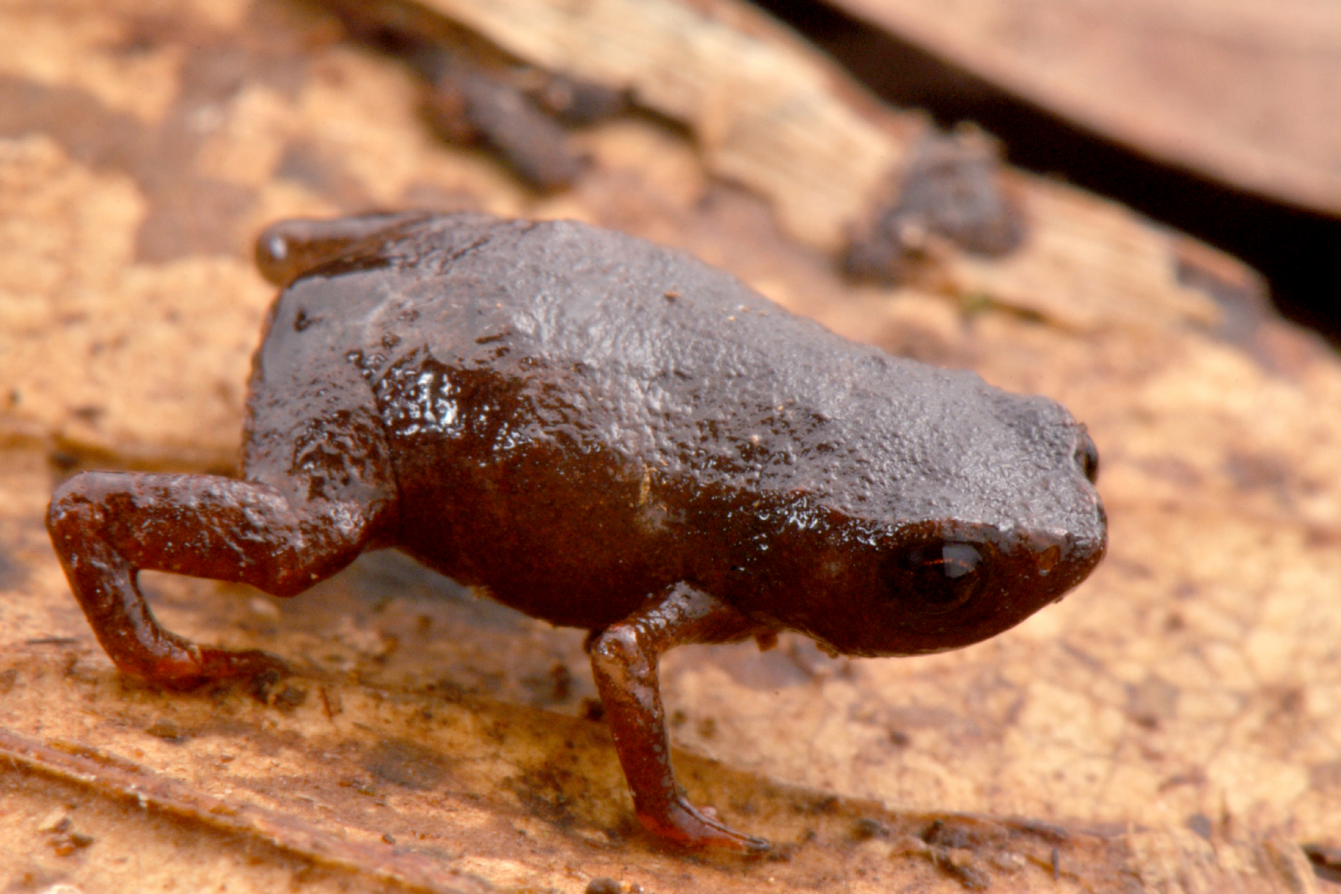
Holotype of *Brachycephalus curupira* in life (MHNCI 10280).

**Figure 8:**
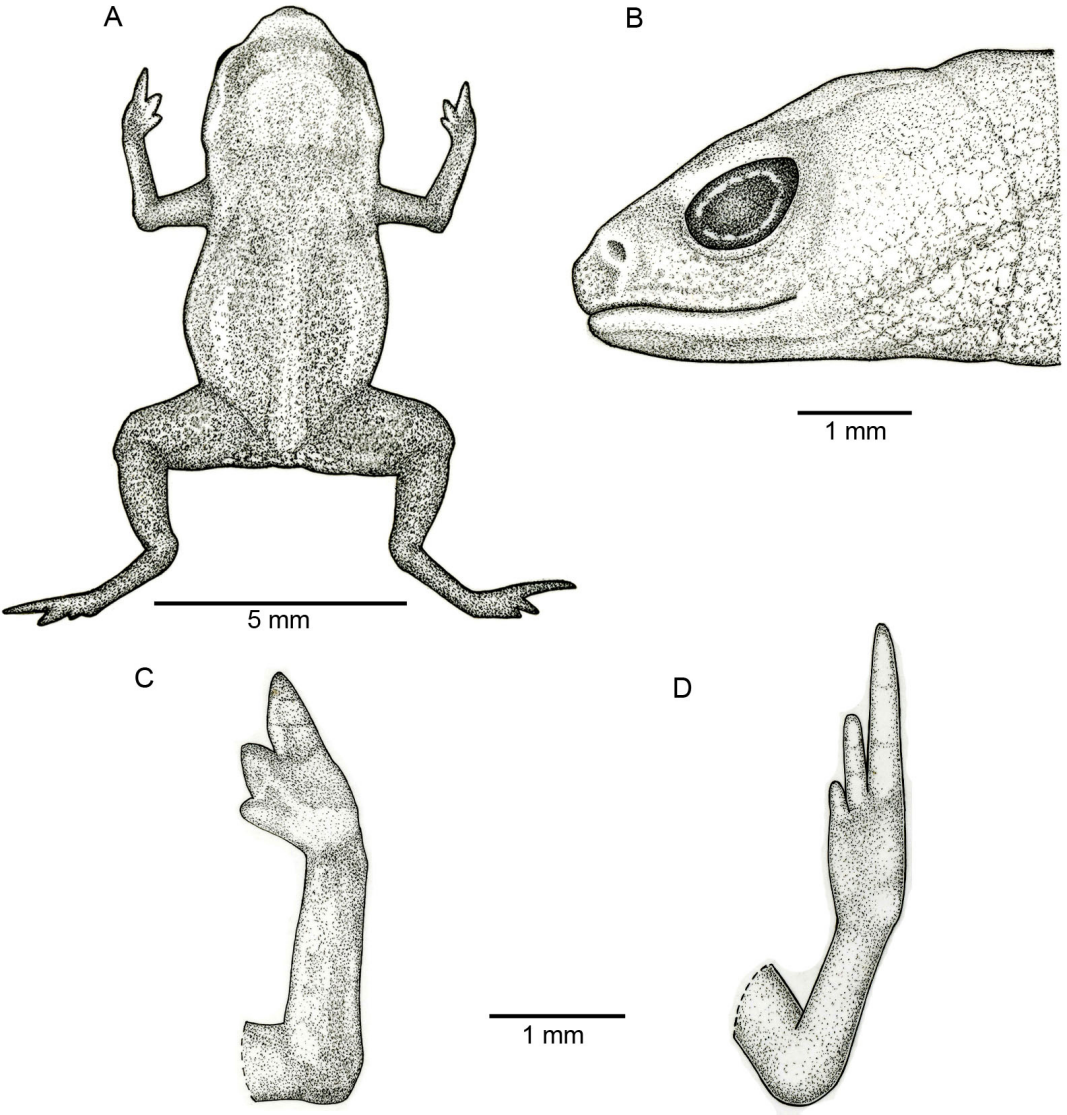
Holotype of *Brachycephalus curupira* (MHNCI 10280). (A) Dorsal view of the body, (B) lateral view of the head, (C) ventral view of right hand, and (D) ventral view of right foot. Drawing by Marcello Brotto.

**Figure 9:**
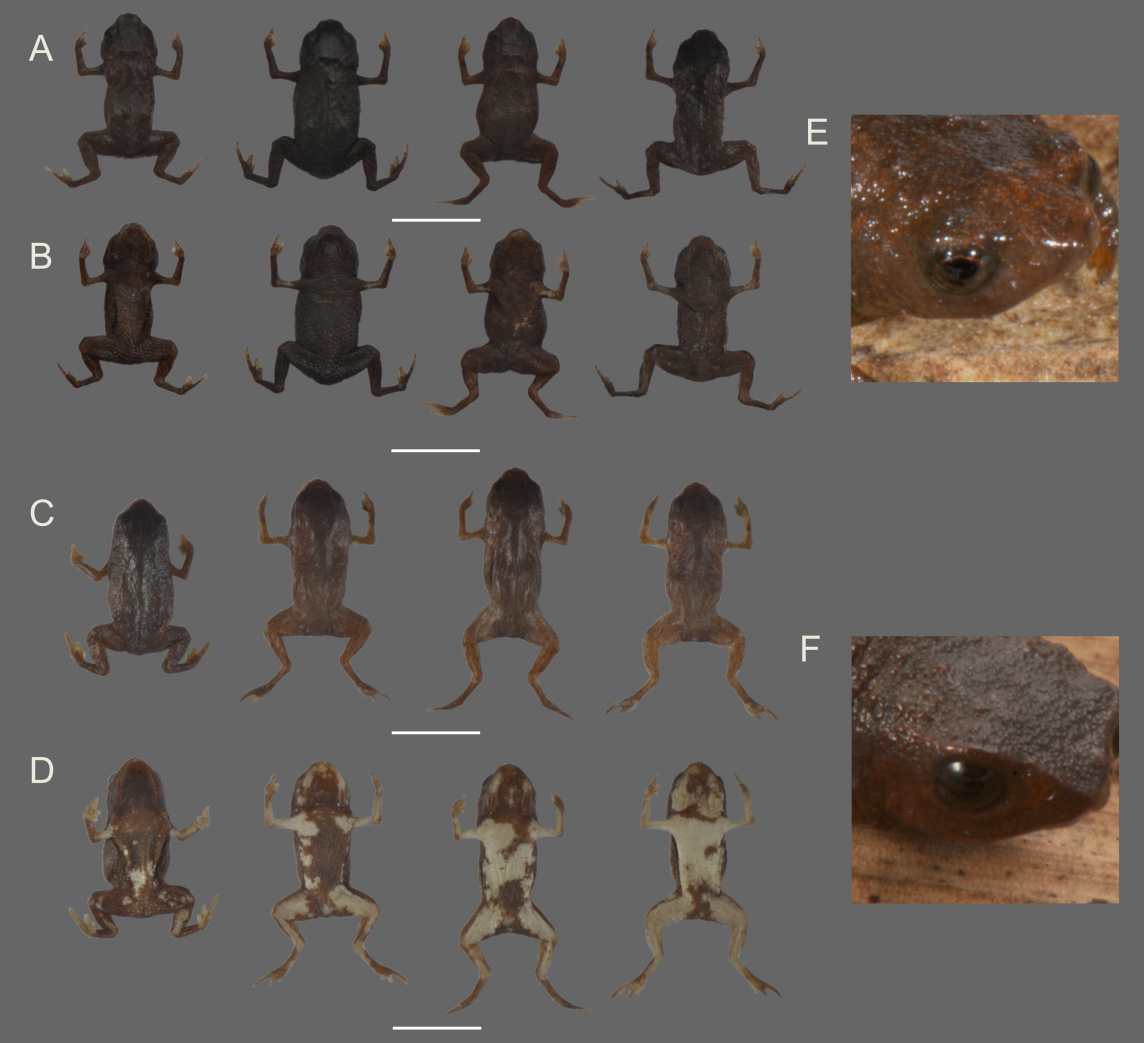
Variation in the coloration of preserved specimens of *Brachycephalus curupira* and *B. brunneus*. *Brachycephalus curupira* (A, dorsal view and B, ventral view, from the left: MHNCI 10292, MHNCI 10287, holotype, MHNCI 10286 paratypes) in contrast with the most morphologically similar congener, *B. brunneus* (C, dorsal view and D, ventral view, from the left: MHNCI 10729-32). Specimens were chosen to represent the most extreme variation in our sample of preserved specimens. Inset: Comparison between eye color in *Brachycephalus curupira* (E, holotype) and *B. brunneus* (F, MHNCI 10733).

**Figure 10:**
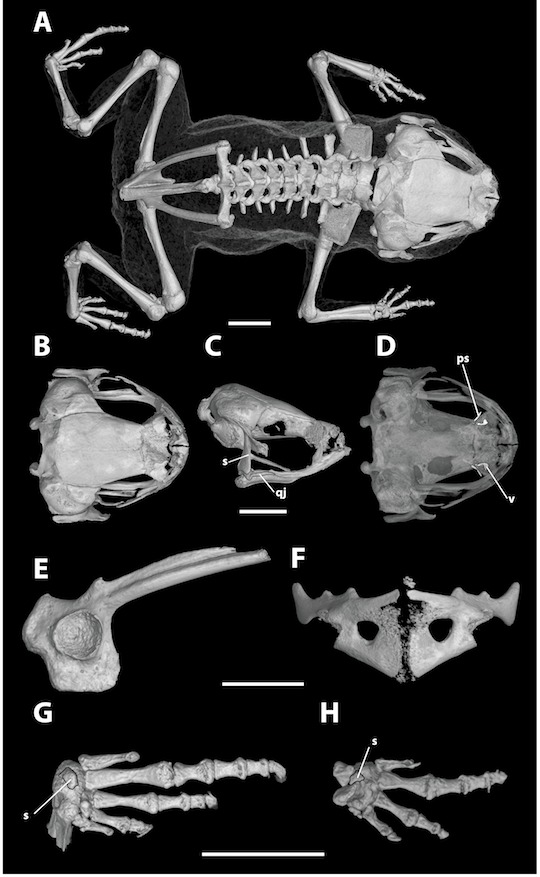
High-resolution computed tomography (CT) scans of a paratype of *Brachycephalus curupira* (MHNCI 10285) showing key osteological features. (A) Dorsal view of the skeleton; (B) dorsal, (C) lateral, and (D) ventral views of the skull (without the lower jaw); (E) ilium in lateral view; (F) pectoral girdle in ventral view; (G) right foot in plantar view; and (H) right hand in palmar view. Abbreviations: n, neopalatine; qj, quadratojugal; s, sesamoid; v, vomer. Scale bars equal 2 mm.

**Figure 11:**
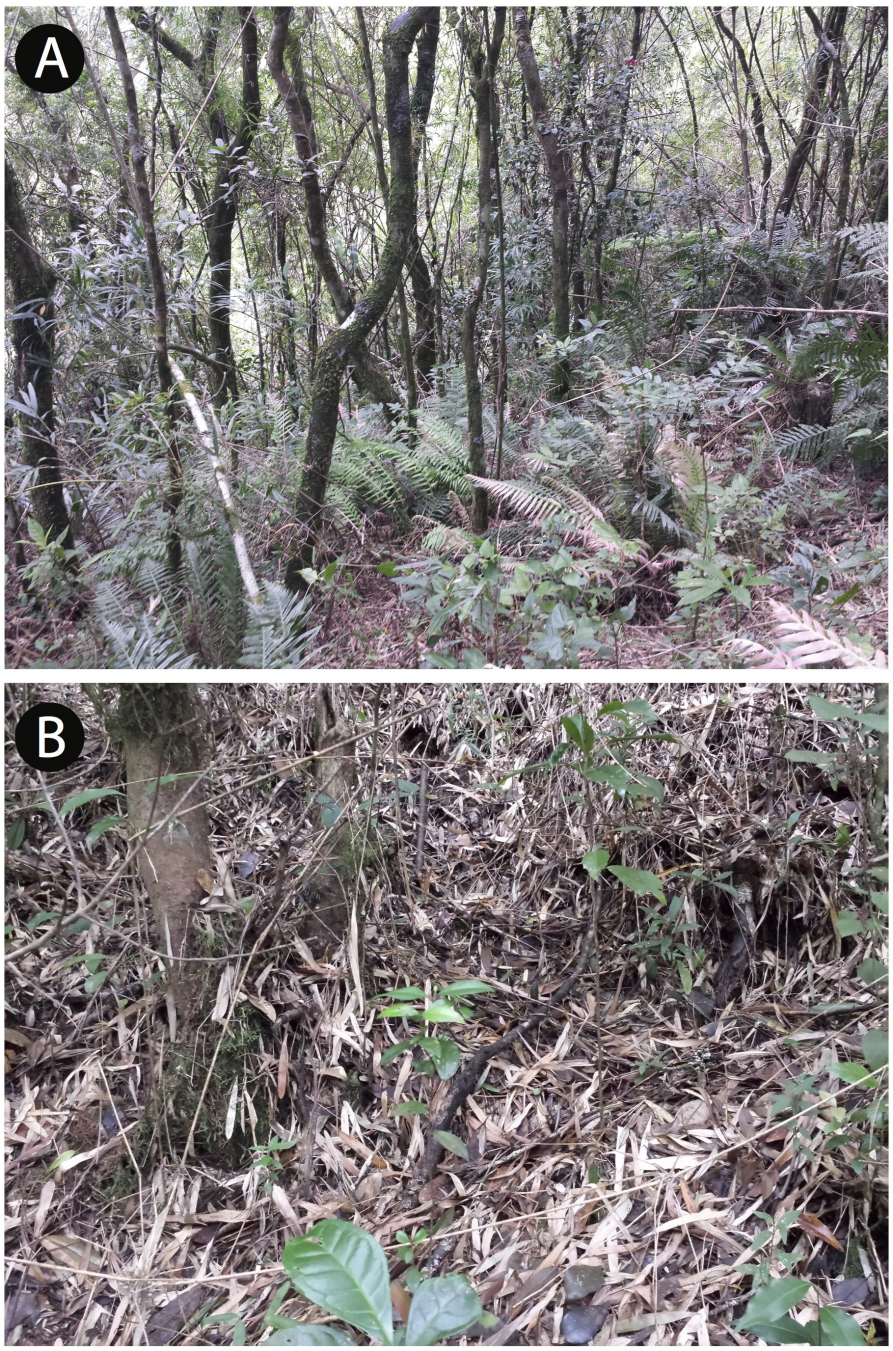
View of the vegetation (A) and the forest floor (B) in the type locality of *Brachycephalus curupira*, at 1,160 m a.s.l. The vegetation is composed of low-canopy montane forest (Floresta Ombrófila Densa Montana), at a rocky location on a steep slope, transitioning into high-montane forest (Floresta Ombrófila Densa Altomontana). Light-colored leaves (B) belong to a bamboo species (*Chusquea* sp.), which in this case is found together with other plant species (as opposed to the situations in which it is dominant).

*Brachycephalus* “sp. 2” (Pie et al., 2013)
*Brachycephalus* “not identified” from “Serra do Salto, Malhada District, municipality of São José dos Pinhais, PR” (Firkowski et al., 2016)
*Brachycephalus* “sp. 6”(Bornschein et al., 2016b)
Urn:lsid:zoobank.org:act:urn:lsid:zoobank.org:act:CB9B7DD6-FE33-4E68-91 E6-C0C1DA579EF6
Figs. 4, 7 –11.

**Holotype.** MHNCI 10280 (Figs. 7, 8, 9A and 9B) adult male, one of a series collected at the Serra do Salto (25°42′07″S, 49°03′44″W; 1,120 m a.s.l.), Malhada District, municipality of São José dos Pinhais, state of Paraná, southern Brazil, on 15 November 2012 by Luiz Fernando Ribeiro and Marcio Roberto Pie.

**Paratypes.** MHNCI 10281 adult female, collected on 26 November 2012 by Luiz Fernando Ribeiro & Marcio Roberto Pie; MHNCI 10282–3 adult males, collected on 26 November 2012 by Luiz Fernando Ribeiro & Marcio Roberto Pie; MHNCI 10284 adult female, collected on 22 January 2016 by Luiz Fernando Ribeiro & Marcio Roberto Pie; MHNCI 10285 adult male, collected on 22 January 2016 by Luiz Fernando Ribeiro & Marcio Roberto Pie; MHNCI 10286 adult male, collected on 27 January 2016 by Luiz Fernando Ribeiro, Marcio Roberto Pie & Marcos Ricardo Bornschein; MHNCI 10287 adult female, collected on 27 January 2016 by Luiz Fernando Ribeiro, Marcio Roberto Pie & Marcos Ricardo Bornschein; MHNCI 10292 adult male, collected on 27 January 2016 by Luiz Fernando Ribeiro, Marcio Roberto Pie & Marcos Ricardo Bornschein. Paratypes were collected between 1,095–1,160 m a.s.l.

**Diagnosis.** *Brachycephalus curupira* is a member of the genus *Brachycephalus* based on diagnostic osteological traits (Fig. 3), including phalangeal reduction, an arciferal pectoral girdle in which the ossified procoracoid and epicoracoid cartilages are fused to the clavicle, coracoid, and scapula, a suprascapula expanded with a prominent cleithrum, and the absence of a sternum (Bornschein et al. (2016a) and modified from Kaplan (2002); Izecksohn (1971); Ford & Cannatella (1993); Ribeiro et al. (2005); Alves et al. (2006); Silva, Campos & Sebben (2007); Fig. 10). *Brachycephalus curupira* is a member of the *B. pernix* group, as defined by Ribeiro et al. (2015), by having a bufoniform body and lacking osteoderms. Within *Brachycephalus*, *B. curupira* is distinguished from all other species by the following combination of characters: (1) body robust and bufoniform; (2) adult size SVL 8.3–10.4 mm; (3) smooth dorsum (Fig. 7); (4) absence of osteoderms; (5) unornamented dermal skull bones that lack co-ossification to the skin; (6) lack of fusion among vertebrae (Fig. 10); (7) presence of distinct neopalatines and vomers; (8) robust squamosal; (9) presence of quadratogjugals; (10) odontoids well-developed on maxillae and weakly developed on premaxillae (Fig. 4); (11) general color (in life) dark brown (Fig. 7); and (8) iris black with golden spots (Fig. 7 and 11A). *Brachycephalus curupira* is the second described species of this species group to be nearly entirely brown (Fig. 7; the other species being *B. brunneus*). However, *B. curupira* differs from *B. brunneus* by having golden spots in the iris (Fig. 9E) rather than being entirely black (Fig. 9F). In preservative, *B. curupira* is often indistinguishable from *B. brunneus* (Fig. 9), particularly given that the preservation often causes the irises to become whitish in both species, thus hampering the visualization of the coloration of the iris. The smooth dorsum of *B. curupira* is similar to that of *B. albolineatus*, *B. brunneus*, *B. coloratus*, *B. leopardus*, *B. pernix*, *B. ferruginus*, *B. izecksohni*, *B. pombali*, and *B. tridactylus* (as opposed to the rugose dorsum of *B. auroguttatus*, *B. boticario*, *B. fuscolineatus*, *B. mariaeterezae*, *B. olivaceus*, *B. quiririensis*, and *B. verrucosus*). *Brachycephalus curupira* lacks osteoderms characteristic of species of the *ephippium* group, as defined by Ribeiro et al. (2015), and its bufoniform shape and larger body size distinguishes it from species in the *didactylus* group, as defined by Ribeiro et al. (2015), which are smaller (SVL = 8–10 mm) and have a leptodactyliform body shape.

**Description of the holotype.** Male with robust, bufoniform body; head slightly wider than long; head length 34% of snout–vent length; snout short with length almost equal to eye diameter, rounded in dorsal and lateral views (Figs. 8A and 8B); nostrils protuberant, directed anterolaterally; canthus rostralis not distinct; lips nearly sigmoid; loreal region slightly concave; eye slightly protuberant in dorsal and lateral views; ED 30% of HL; tympanum indistinct; vocal sac not expanded externally; tongue longer than wide, with posterior half not adherent to floor of mouth; choanae relatively small and round; vomerine teeth absent.

Upper arm and forearm relatively slender, upper arm approximately as long as forearm; tip of fingers I and II slightly rounded, tip of Finger III pointed; relative lengths of fingers IV < I < II < III; subarticular tubercles and inner and outer metacarpal tubercles absent; legs short, moderately robust; thigh length 35% of snout–vent length, crus length 91% of thigh length; toes II–IV short, relatively distinct; toes I and V not visible externally; relative length of toes II < III < IV; subarticular tubercles and inner metatarsal tubercles absent; outer metatarsal tubercle distinct, large, ovoid.

Dorsum smooth, without osteoderms; head, arms, legs and flanks smooth; belly and ventral surface of thighs with small, circular to ovoid glands; chin smooth.

**Coloration of the holotype.** In life, dorsum, head, dorsal and ventral region of arms, sides of the body, and legs dark brown (Fig. 7). Iris black with golden spots. In preservative, the overall dark brown coloration in maintained, with the tips of fingers and toes becoming pale cream (Fig. 9).

**Measurements of holotype (in mm).** SVL = 9.7, HL = 3.3, HW = 3.7, ED = 1.1, ND = 0.2, IOD = 2.0, IND = 1.2, END = 0.5, THL = 3.4, CL = 3.1.

**Variation in the type series. Variation in the type series.** Measurements (in mm) of 10 adult males are (mean ± SD, with range in parentheses): SVL = 9.9 ± 0.7 (8.9–10.7); HL = 3.2 ± 0.2 (2.9–3.6); HW = 3.7 ± 0.2 (3.4–4.0); ED = 1.2 ± 0.1 (1.0–1.3); ND = 0.2 ± 0.0 (0.1–0.2); IOD = 1.9 ± 0.1 (1.7–2.1); IND = 1.0 ± 0.1 (1.0–1.2); END = 0.5 ± 0.0 (0.4–0.6); THL = 3.4 ± 0.2 (3.1–3.7); CL = 3.1 ± 0.2 (2.9–3.3). Measurements of four adult females are: SVL = 10.1 ± 1.7 (8.3–12.3); HL = 3.3 ± 0.4 (2.9–3.7); HW = 3.9 ± 0.4 (3.4–4.4); ED = 1.2 ± 0.1 (1.1–1.4); ND = 0.2 ± 0.0 (0.2–0.2); IOD = 1.9 ± 0.1 (1.8–2.1); IND = 1.1 ± 0.1 (1.1–1.2); END = 0.5 ± 0.1 (0.5–0.6); THL = 3.7 ± 0.5 (3.1–4.3); CL = 3.1 ± 0.2 (2.9–3.4). Although the entire body tended to be completely dark brown, some individuals showed a few small yellow dots on their ventral region, which is present even in preserved specimens, but as pale cream. Some individuals display a faint, darker “X”-shaped mark on the anterior region of the dorsum, which remains visible in preserved specimens. The dark brown coloration is maintained in preserved specimens, with the tips of fingers and toes becoming pale cream, but with little variation among individuals (Fig. 9).

**Description of general osteology.** Based on Micro-CT scan of MHNCI 10285 (Fig. 10), *Brachycephalus curupira* is broadly similar to both the above species and others in the genus. The skull is compact, slightly wider than long, and lacking ornamented dermal roofing bones. The well-developed parotic plate is synostosed to the frontoparietal approximately at the location of a prominent groove for the occipital artery. The nasals, frontoparietals, prootics, and exoccipitals are synostosed. The sphenethmoid is densely mineralized but no synostosed to adjacent elements. The margins of the optic fenestrae and prootic foramina are less well defined in comparison to *B. coloratus* above. The premaxillae are broad, widely separated, and have weakly developed odontoids; each has a robust pars dentalis, and a robust alary process that is taller than wide and widely separated from the adjacent nasal. In ventral view, the maxillae are weakly curved and bear well-developed odontoids along their anterior half. The quadratojugals are prominent with a broad articulation with the maxillae. The pterygoids are slender, each with a long anterior ramus that approaches but does not articulate with the adjacent maxilla, a short posterior ramus approaches the ventral ramus of the squamosal, and a short and broad medial ramus approaches the prootic. Distinct vomers and neopalatines are present. The vomer lacks a dentigerous process and bears distinct though very short pre- and postchoanal processes, giving it a broad boomerang shape. Small, curved sphenethmoids are present at the anterior margin of the nasal capsule. The parasphenoid is broad and robust. The squamosals are robust, and each bears a large rectangular zygomatic ramus and a long and slender posterior ramus that has a broad connection to the prootic. Each fenestra ovalis is covered entirely by a robust, ossified operculum; a stapes (or columella) is not present. The posteromedial processes of the hyoid are ossified and slender. The arytenoid cartilages are not obviously mineralized.

There are eight distinct, procoelous, non-imbricating presacral vertebrae that are not fused to one another. The atlas lacks transverse processes and has widely separated cotyles. The sacrum is procoelous with stout transverse processes. A sesamoid at the distal transverse process of the sacrum is present at its articulation with the ilium. The urostyle is relatively stout with a prominent dorsal ridge (though not as robust as in *B. coloratus* above) that decreases in height posteriorly.

The robust pectoral girdle is arciferal and formed by the synostosis of the clavicle, coracoid, scapula. Because the epicoracoid and procoracoid cartilages are relatively weakly mineralized (compare to *B. coloratus* above), the clavicle and coracoid can be distinguished. The scapula is stout and bears a prominent anterior process. A bony sternum and omosternum are not present.

The pelvic girdle is a robust element composes of synostosed ilium, pubis, and ischium. The circular acetabulum is completely ossified with well-defined margins. The shaft of the ilium is straight in lateral view and weakly bowed in dorsal view, and bears both a prominent dorsal protuberance and a well-defined dorsal crest. There is a broad ventral acetabular expansion comprised of both the ilium and ossified pubis.

The forearm is shorter than the humerus. The distal carpals (Element Y and II–V) are fused. The radiale and ulnare are large and subequal in size. The phalangeal formula for the manus is 1–2–3–1 and there is both a single ossified prepollex and a small palmar sesamoid. The tips of the terminal manual phalanges are generally blunt. The tibiofibula and femur are similar in length. There are two large distal tarsals. The phalangeal formula for the pes is 0–1–3–4–0 and there is a plantar sesamoid but not an obvious distinct prehallux. The tips of the terminal pedal phalanges are arrow-shaped in digits II–IV; there are no phalanges for digits I and V.

Tomograms (tif format) and shape files (stl format) of the studied specimen are freely available at http://morphosource.org/Detail/SpecimenDetail/Show/specimen_id/4220.

**Etymology.** The specific epithet is a noun in apposition and refers to the homonymous mythical character in Brazilian folklore whose aim is to protect the forests. Although usually portrayed as a red-headed boy with feet pointing backwards, the curupira becomes invisible and produces sounds that confuse those walking in his forests. This confusion is a fitting description of our situation while trying to locate calling males of this elusive species (see Remarks below).

**Distribution, habitat and abundance.** The species is known from the type locality, where it was found in three types of vegetation: montane forest (Floresta Ombrófila Densa Montana; Fig. 11A), montane forest in a transition to high-elevation montane forest (Floresta Ombrófila Densa Altomontana), and in a secondary forest, formed in areas previously covered by montane forest. In all of these habitats, it is highly abundant a bamboo species (*Chusquea* sp.), especially in secondary forests. The estimate of its ”extent of occurrence” (*sensu* IUCN, 2012) is 2,211 ha (Bornschein et al., 2016b).

We recorded the *Brachycephalus curupira* calling throughout the day under the leaf litter (Fig. 11B), but with more intense vocal activity in the morning and later in the day. In periods of the day with warmer temperatures (c. 11 h–16 h), vocal activity was strongly reduced. The species is abundant, yet extremely difficult to locate and capture. Indeed, in comparison to all other species of *Brachycephalus* that we have investigated in southern Brazil, we experienced our worst capture success rate (∼0.5 specimen per researcher per day of field work) for *B. curupira*. This difficulty is due to the highly cryptic coloration of the species that closely matches the coloration of the litter, as well as the difficulty in determining the precise location of the callings individuals. Collected individuals were found beneath the leaf litter just above the soil.

*Brachycephalus curupira* had a patchy distribution throughout approximately 700 m of transect along trails and within the forest in the type locality. It is possible that the abundance of the species is regulated by the quality of the leaf litter, which in turn is partly affected by vegetation and slope. We found higher abundance (one individual per ∼2–3 m^2^) in areas dominated by *Chusquea* sp., whereas lower abundances (one individual each ∼6–7 m^2^ and ∼15–16 m^2^) were estimated in sites where *Chusquea* sp. was less common.

**Figure 12:**
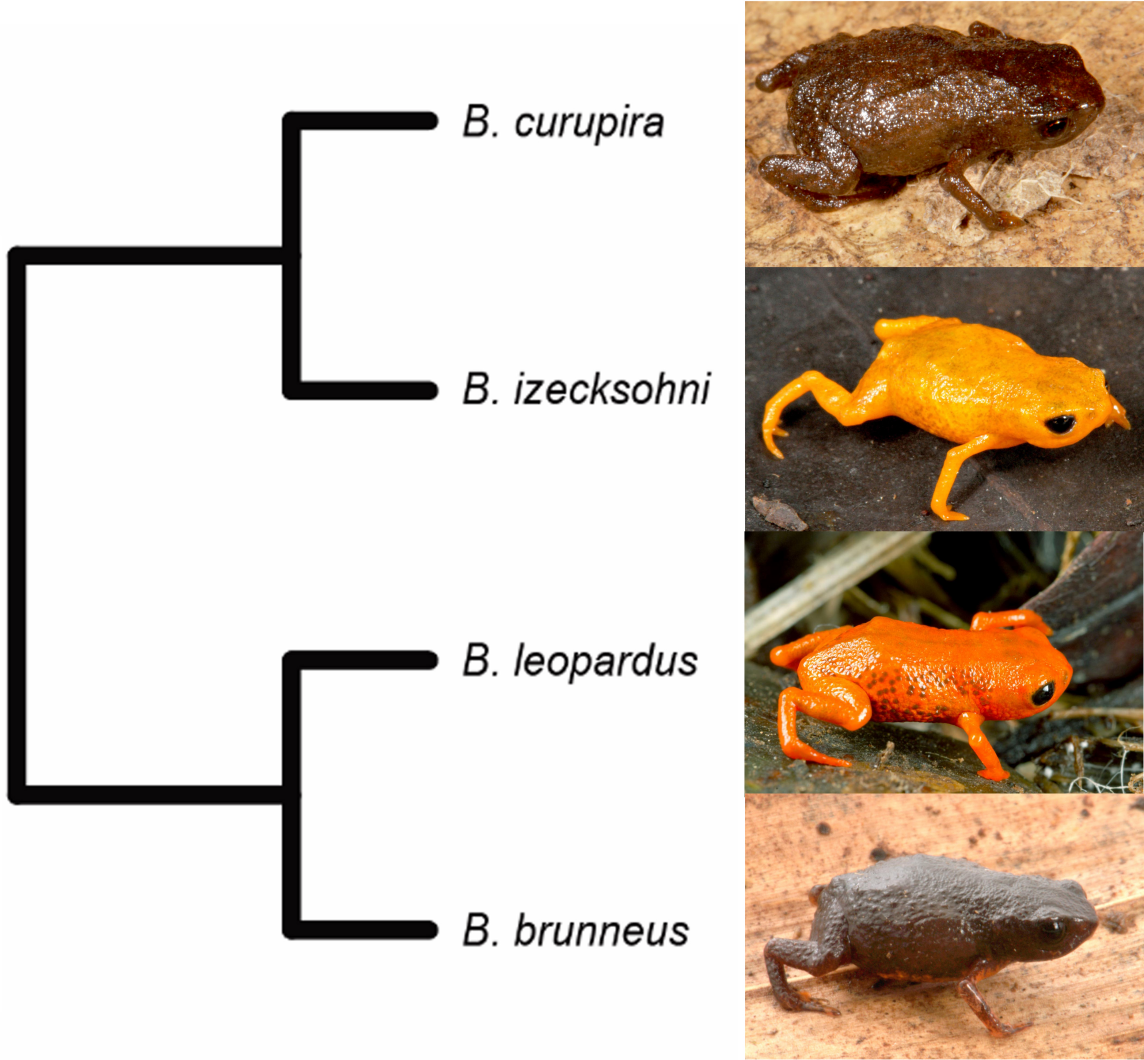
Phylogenetic relationships between *Brachycephalus curupira* and closely related species (modified from the phylogeny of Firkowski et al. (2016) to include only described species). Photographs by Zig Koch (*B. leopardus*) and LFR (remaining species).

**Remarks.** The validity of *Brachycephalus curupira* was previously established with molecular species delimitation methods (Firkowski et al., 2016), which is consistent with our morphological diagnosis. In particular, of all species that have already been formally described, *B. curupira* is most closely related to *B. izecksohni*, a yellow species, whereas the most morphologically similar species to *B. curupira*, the brown *B. brunneus*, is more closely related to *B. leopardus* (Fig. 12; Table 1). Another characteristic that distinguishes the great majority of the specimens of *B. curupira* from *B. brunneus* is the presence of yellow irregular regions of varying extent on the ventral side of the latter, whereas the great majority of the specimens of *B. curupira* are completely brown. Only rare specimens of *B. curupira* showed some small yellow dots on ventral side, becoming similar to the rare specimens of *B. brunneus* with only little yellow (to the best of our there is no known specimen of *B. brunneus* that is completely brown, contrary to the assertion by Ribeiro et al. (2005)). In preservative, the yellow of live specimens of *B. brunneus* becomes pale cream and this characteristic also can be used to distinguish them from the specimens of *B. curupira* without yellow on the ventral surface, which maintain the overall dark brown coloration in preservative.

**Table 1:** Uncorrected p-distances within and among species of *Brachycephalus* related to *B. curupira* based on sequence data from Firkowski et al. (2016) for the 16S mitochondrial gene. The main diagonal indicates the average standard deviation of intraspecific distances and the corresponding sample sizes, whereas off-diagonals indicate average standard deviations of interspecific distances.

Species	*B. curupira*	*B. brunneus*	*B. leopardus*	*B. izecksohni*
*B. curupira*	0 ± 0 (*N* = 5)			
*B. brunneus*	0.003 ± 0.0008	0.0022 ± 0.003 (*N* = 10)		
*B. leopardus*	0.0086 ± 0.0006	0.0115 ± 0.0026	0.00038 ± 0.0008 (*N* = 11)	
*B. izecksohni*	0.0029 ± 0.001	0.0066 ± 0.0013	0.0115 ± 0.0012	0.0012 ± 0.001 (*N* = 11)

The polygon of 2,211 ha of the extent of occurrence of the species in its type locality (Bornschein et al., 2016b) disregarded some inhospitable areas, such as 80 ha of *Pinus* spp. plantations, 40 ha of exposed rocks, and other areas occupied by other cultivations, roads, residences and granite mining (MRB, pers. obs., 2015). Other impacts that might also take place in the extent of occurrence of the new species include vegetation clearing below power lines, forest fires, and selective logging. Areas with *Pinus* spp. plantations may be expanded up to regions of exposed rocks that are common in the region, which could prevent forest recovery from secondary succession to a forest that is suitable for the occurrence of the new species. *Pinus* spp. also disturb local microclimatic conditions and soil quality (e.g., Pacheco et al., 2009). The range of the species was estimated as extent of occurrence rather than “area of occupancy” (*sensu* IUCN, 2012), although some landscapes were disregarded because the species does not necessarily occur across the entire mapped polygon (see above). We suggest that the species should be considered as Data Deficient until we can make more accurate evaluations after further field research.

**Figure 13:**
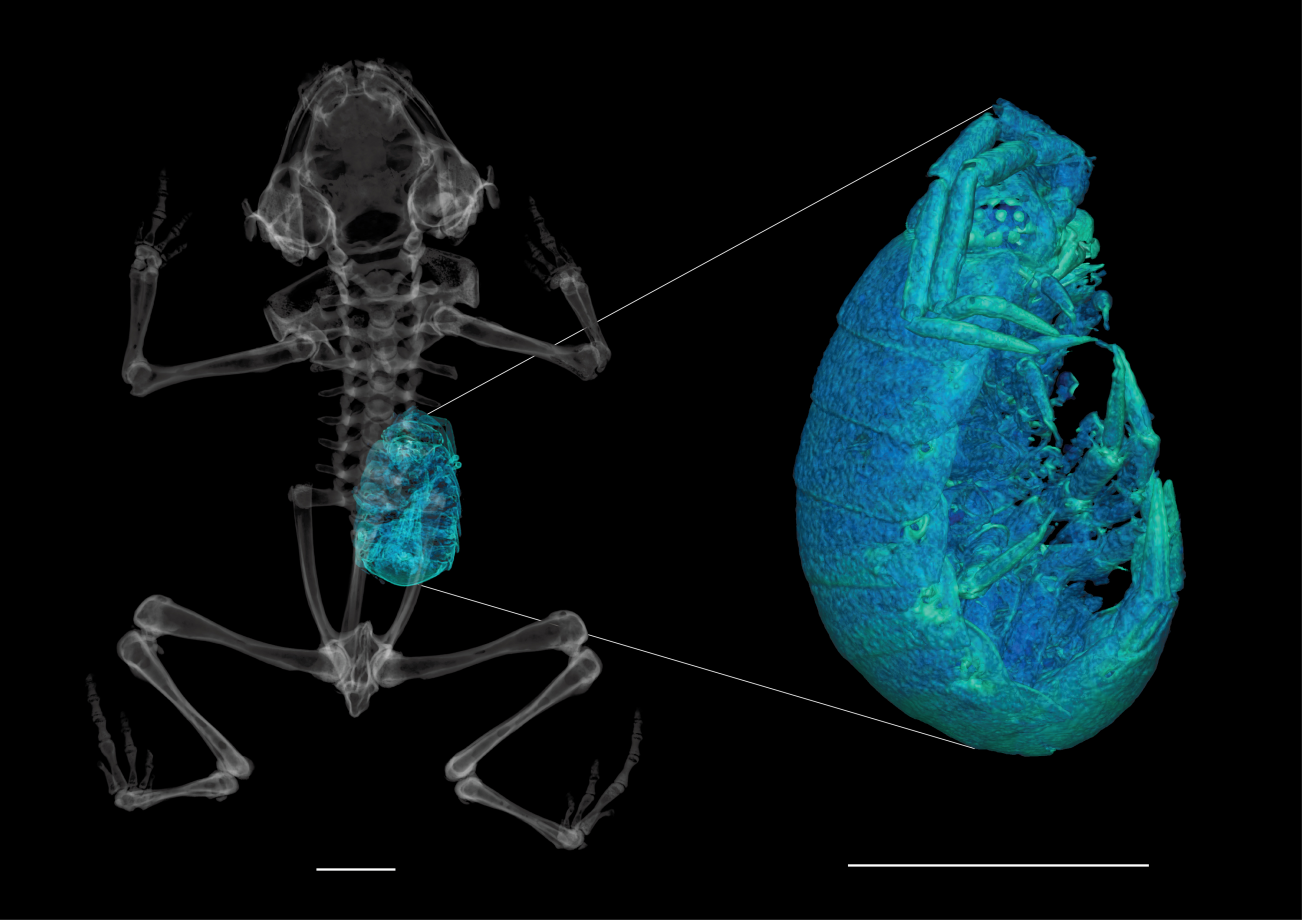
High-resolution CT scan of paratype of *Brachycephalus curupira* (MHNCI 10285) revealing a single isopod (enlarged at right) in the stomach. Scale bars equal 2 mm.

Our CT scan of a paratype (MHNCI 10285) revealed that this specimen contained a single large isopod in its stomach (Fig. 13). Other species of *Brachycephalus*, such as *B. brunneus* (Fontoura, Ribeiro & Pie, 2011), are known to consume isopods in addition to other small leaf litter arthropods.

## Discussion

**Figure 14:**
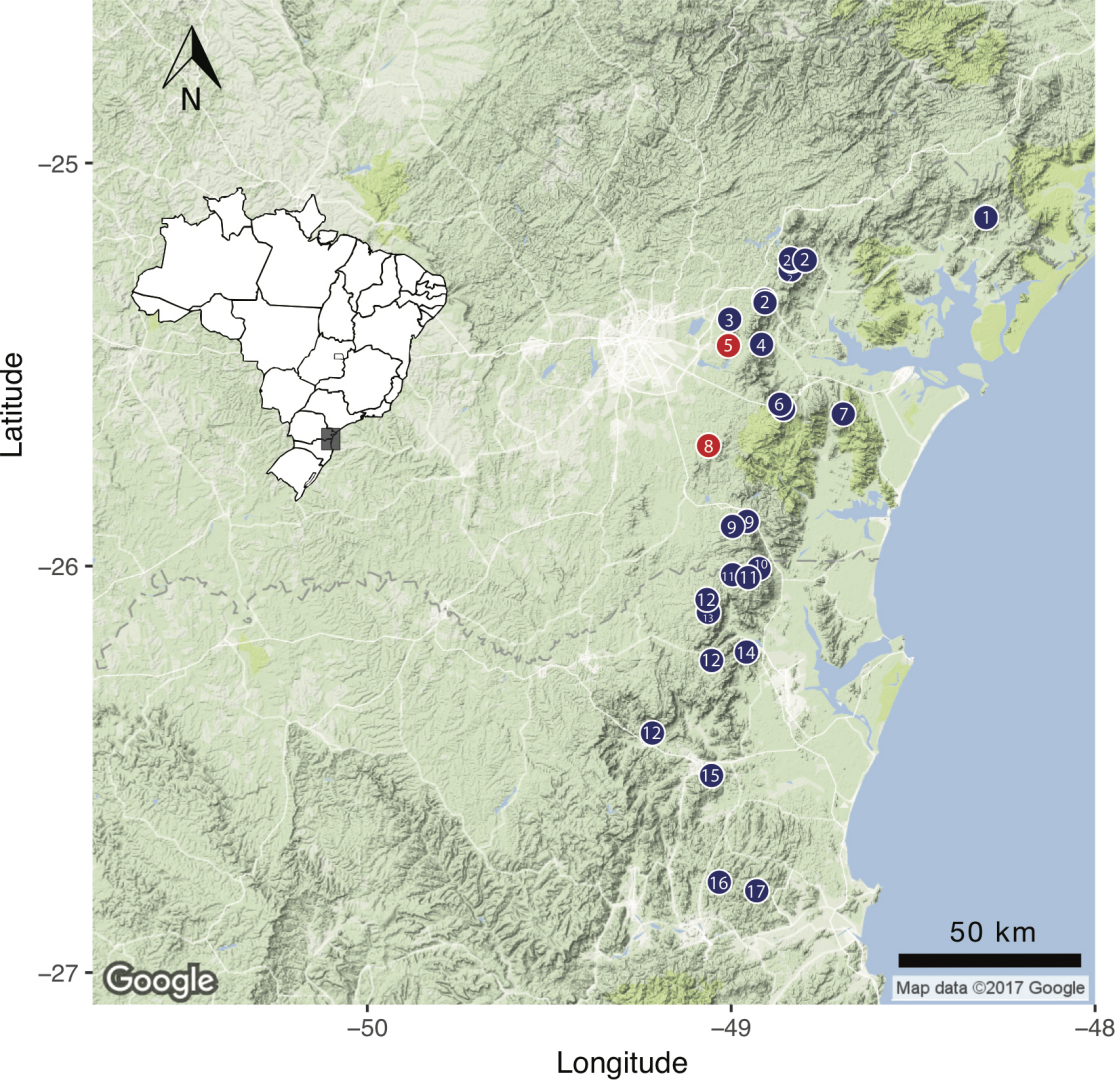
Geographical distribution of all described *Brachycephalus* species of the *B. pernix* species group, based on Bornschein et al. (2016a) and Bornschein et al. (2016b) and the present study. (1) *B. tridactylus*, (2) *B. brunneus*, (3) *B. pernix*, (4) *B. ferruginus*, (5) *B. coloratus*, (6) *B. pombali*, (7) *B. izecksohni*, (8) *B. curupira*, (9) *B. leopardus*, (10) *B. auroguttatus*, (11) *B. quiririensis*, (12) *B. olivaceus*, (13) *B. mariaeterezae*, (14) *B. verrucosus*, (15) *B. albolineatus*, (16) *B. boticario*, and (17) *B. fuscolineatus*. The geographical locations of the new species are indicated in red.

The phylogenetic relationships within the *B. pernix* group of *Brachycephalus* presented by Firkowski et al. (2016) based on molecular data revealed that the clade including the cryptic *B. curupira* and *B. brunneus* also includes two brightly colored species (*B. izecksohni* and *B. leopardus*, Fig. 12). Regardless of the ancestral coloration of the clade, we can infer that there have been changes in the presence of bright coloration patterns during its diversification, either leading to the loss of bright coloration or by its evolution from a cryptic ancestor. Given that the current model for the diversification of *Brachycephalus* in southern Brazil suggests that species evolved in allopatry (Bornschein et al., 2016b; Firkowski et al., 2016), it is interesting to note that nearly all species described to date in this clade can be promptly diagnosed by coloration of the body, which suggests that coloration is strongly selected in this clade, even though they are distributed allopatrically (Fig. 14). *Brachycephalus curupira* is the first described species of the *B. pernix* group that cannot be diagnosed by coloration alone (only by iris coloration). This is not surprising, given that there are few possibilities for cryptic coloration in a litter-dwelling species, as opposed to their brightly colored congeners.

The geographical distribution of *Brachycephalus coloratus* is a new example among the few known cases of distinct species occurring in a single mountain range (“*serras*”). In this case, *B. coloratus* occurs in the Serra da Baitaca together with *B. pernix*.

## Appendix. Examined specimens

*Brachycephalus albolineatus*. SANTA CATARINA: Morro Boa Vista, on the border between the municipalities of Jaraguá do Sul and Massaranduba MHNCI 10290 (holotype), MHNCI 10295–10300, MNRJ 90349 (all paratypes).

*Brachycephalus auroguttatus*. SANTA CATARINA: Pedra da Tartaruga, municipality of Garuva DZUP 375 (holotype), DZUP 373–4, 376–85, 387–89 (all paratypes).

*Brachycephalus boticario*. SANTA CATARINA: Morro do Cachorro, on the border between the municipalities of Blumenau, Gaspar, and Luiz Alves DZUP 440 (holotype), DZUP 414–5, 438–9, 444–5, 459 (all paratypes).

*Brachycephalus brunneus*. PARANÁ: Caratuva, Serra dos Órgãos, municipality of Campina Grande do Sul MHNCI 1919–20, MNRJ 40289–91 (paratypes).

*Brachycephalus didactylus*. RIO DE JANEIRO: municipality of Engenheiro Paulo de Frontin ZUEC 10825, MZUSP 94621; Sacra Família do Tinguá, municipality of Engenheiro Paulo de Frontin ZUEC 1132–3, MZUSP 13613–20, 64810–1, 94621.

*Brachycephalus ephippium*. RIO DE JANEIRO: Parque Nacional Serra dos Órgãos MZUSP 104140–7; Vale de Revolta MCZ A–108655. SÃO PAULO: municipality of Cotia MHNCI 2611–16.

*Brachycephalus ferruginus*. PARANÁ: Olimpo, Serra do Marumbi, municipality of Morretes MHNCI 125, 128.

*Brachycephalus fuscolineatus*. SANTA CATARINA: Morro do Baú, municipality of Ilhota DZUP 159 (holotype), DZUP 158, 160, 401–5 (all paratypes).

*Brachycephalus hermogenesi*. SÃO PAULO: Ubatuba ZUEC 9715 (holotype), ZUEC 9716–25 (paratypes).

*Brachycephalus izecksohni*. PARANÁ: Torre da Prata, Serra da Prata, on the border between the municipalities of Morretes, Paranaguá, and Guaratuba CFBH 7381–2, 7384 (all paratypes).

*Brachycephalus leopardus*. PARANÁ: Serra do Araçatuba, municipality of Tijucas do Sul DZUP 490 (holotype), DZUP 478–89, 491–2 (all paratypes); Morro dos Perdidos, municipality of Guaratuba DZUP 274–83.

*Brachycephalus mariaeterezae*. SANTA CATARINA: Reserva Particular do Patrimônio Natural Caetezal, top of the Serra Queimada, municipality of Joinville MHNCI 9811 (holotype), DZUP 372, 393–9 (all paratypes).

*Brachycephalus nodoterga*. SÃO PAULO: Reserva Biológica Tamboré, municipality of Santana de Parnaíba MZUSP 147711–6.

*Brachycephalus olivaceus.* SANTA CATARINA: base of the Serra Queimada, municipality of Joinville MHNCI 9813 (holotype), DZUP 371 (paratype); Castelo dos Bugres, municipality of Joinville MHNCI 9814–8 (paratypes); Morro do Boi, Municipality of Corupá MHNCI 10288–9.

*Brachycephalus pernix*. PARANÁ: Anhangava, Serra da Baitaca, municipality of Quatro Barras MNRJ 17349 (holotype), CFBH 2597–8 (paratypes), MHNCI 1818–9, 3000–4 (all paratypes), MHNCI 1820, ZUEC 9433–7 (paratypes), DZUP 539–55.

*Brachycephalus pitanga*. SÃO PAULO: SP 125, municipality of São Luís do Paraitinga DZUP 407–9.

*Brachycephalus pombali.* PARANÁ: Morro dos Padres, Pico da Igreja, municipality of Guaratuba CFBH 8042 (holotype), 8043–53 (paratypes).

*Brachycephalus quiririensis*. SANTA CATARINA: Serra do Quiriri, municipality of Campo Alegre DZUP 172 (holotype), DZUP 171, 173–6, 524–30 (all paratypes).

*Brachycephalus sulfuratus*. SÃO PAULO: base of the Serra Água Limpa, municipality of Apiaí DZUP 362. PARANÁ: Caratuval, near the Parque Estadual das Lauráceas, municipality of Adrianópolis DZUP 139; Corvo, municipality of Quatro Barras DZUP 150–7; Fazenda Thalia, municipality of Balsa Nova DZUP 221–4; Mananciais da Serra, municipality of Piraquara MHNCI 10302; Recanto das Hortências, municipality of São José dos Pinhais DZUP 463; Salto do Inferno, Rio Capivari, municipality of Bocaiúva do Sul MHNCI 9800.

*Brachycephalus verrucosus*. SANTA CATARINA: Morro da Tromba, municipality of Joinville MHNCI 9819 (holotype), MHNCI 9820 (paratype), DZUP 464–78 (paratypes).

*Brachycephalus tridactylus*. PARANÁ: Serra do Morato, Reserva Natural Salto Morato, municipality of Guaraqueçaba DZUP 493–7, MHNCI 10294, 10729–30.

## Additional Information and Declarations

### Data Availability

The following information was supplied regarding data availability: Morphosource: http://morphosource.org/Detail/SpecimenDetail/Show/specimen_id/4219 and http://morphosource.org/Detail/SpecimenDetail/Show/specimen_id/4220.

### New Species Registration

The following information was supplied regarding the registration of a newly described species: *Brachycephalus coloratus*: urn:lsid:zoobank.org:act:D45D8C67-0F74-459E-8FC6-048748ED254B. *Brachycephalus curupira*: urn:lsid:zoobank.org:act:CB9B7DD6-FE33-4E68-91E6-C0C1DA579EF6 Publication LSID: urn:lsid:zoobank.org:pub:09776B0B-A24F-4D13-BC93- DC9CE26C40D9
